# A Treatise on Rheumatic Gout, or Chronic Rheumatic Arthritis of All the Joints

**Published:** 1859-03

**Authors:** 


ART. IV.—*A Treatise on Rheumatic Gout, or Chronic Rheumatic Arthritis of all the Joints.* By ROBERT ADAMS, M.D., A.M., M.R.I.A., ex-President of the College of Surgeons in Ireland, and of the Pathological Society; Surgeon to the Richmond Hospital, Dublin. Illustrated by Wood-cuts, and an Atlas of Plates. London: Churchill. 1857. 8vo., pp. 352; 11 Plates.

WE were at first misled by the title of this work, and fancied that it related principally to the internal pathology of gout and rheumatism, and the mixture or confusion of those affections commonly alleged to be designated by the term rheumatic gout. We were not prepared to find it leading us through investigations most generally met with as the objects of surgery.

Undoubtedly, an elaborate examination of the organic changes exhibited, is matter quite of the cast which has, during many years, possessed so deep an interest for internal pathologists; and the chirurgical aspect of the book is the simple consequence of the situation of the lesions. The work is based upon a profound and elaborate study of the visible changes in the joints. Yet we might have expected something more. Not a word is said, at least beyond mere definitions of words, of the causes, nature, or treatment of either gout or rheumatism in its early or middle stage. There is a total absence of microscopical considerations; and nearly the same may be said in regard to chemical views; and no mention whatever is made of the concomitant or consequent affections of the endocardium, or valves of the heart. Besides all this, our author's own cases are entirely confined to rheumatic subjects; and the introduction of the word gout into the title-page at all, is only justified by his abstracts of the observations of Haygarth and Sir Benjamin Brodie.

We are thus tempted to doubt the propriety of the title. Arthritis is, no doubt, etymologically correct in the case, and certainly includes gout when used in the works of Hippocrates; but is it just to assume the present meaning of the word to be such as Dr. Adams has implied? We believe the present usage of the profession to be decidedly in favor of confining the employment of the term gout, and with it, of its translation, arthritis, to the disease which begins with visceral disorder, and produces, in an evident manner, an excess of uric acid in the secretions, and in the nodose deposits formed by the morbid process in the joints. Not to speak of the less recent modern authorities, such is certainly to be inferred from an inspection of the half dozen later works to which we have referred with this object, including an earlier edition of Dr. Wood's Practice, and the volumes of Drs. S. H. Dickson, Watson, and Williams, and among the Germans, those of Canstatt. It is true that Dr. Wood, in his recent edition, opens this question again, and speaks of it as undecided, following in his classification the usage of Dr. Adams; whose work, however, he states he has not had an opportunity of examining. The disease described by Dr. Adams appears to occur almost exclusively among the poor, all of them being persons exposed to cold, namely, the Irish laborers. Of the propriety of the term rheumatic inflammation of the small joints, there can perhaps be no real doubt. To that of arthritis, used as a translation of gout, in which sense it will certainly be taken by the great mass, and is taken by Dr. Wood in the passage referred to, we cannot withdraw our objection that it keeps up confusion and doubt in a case where all the clearness and positiveness that are attainable are certainly earnestly demanded by the interests of mankind. It is not uncommon to hear words, when subjects of discussion, spoken of as trifles. They are not always trifles; they relate to things, and are sometimes important things. What objection lies to the expression so popular with the French schools, articular rheumatism? Were we called upon, we should have defined the subject of the present valuable work as the Anatomical Consequences of Chronic Inflammation of the Joints from Internal Causes, such as Rheumatism, and, if the author pleases, Gout.

The first chapter of Dr. Adams's work is a notice of the memoirs on this affection, published from the time of Dr. Haygarth down.

The *causes* of the disease are generally constitutional; exposure to cold, humidity, and night air, after the operation of depressing agents on the body; cold after the body has been much heated by hard and excessive exertion; and being kept standing for a long time in cold water. Of local causes, Dr. Adams cites from Dupuytren the occurrence of a similar affection in the wrist-joint from habitual excessive straining of that joint, as in printers' pressmen; and from Haygarth, a case which originated in a sprain. In his own experience, he has seen instances which occurred in the hip-joint, from excessive exertion of the part; and in the shoulder and hip he has witnessed the same phenomena from severe concussions. The writer of this believes that he can add an instance occurring in the ultimate joints of the fingers from continued exposure to severe cold. There had been one hereditary antecedent in the case.

Dr. Haygarth found the disease principally prevalent among persons who lived luxuriously, and used but little exercise. It appeared to him to be more connected with the use of an excess of animal food, than with that of fermented liquors. Dr. Haygarth found frequent gouty deposits; and the outbreak of the disease was frequently preceded by those gastric disorders which are usually considered the forerunners of gout in its regular form. The experience of Dr. Adams is directly the reverse in these particulars.

*Symptoms.*—The disease is essentially chronic, never being accompanied by much heat in the part. The swelling at first is large, rounded, and not hard, being in general partly composed of an augmented synovial secretion. The joint is stiffened, and limited in its motion, with little or no pain. By long continuance of the disease, the stiffness increases, the rounded swelling diminishes, the fluctuation of synovia disappears, and the nodosity becomes visible. Spasms of the corresponding muscular fibres frequently, or even generally, accompany the process: this again being in opposition to the experience of Sir B. Brodie.

The nodosity, which so much fixed the attention of Dr. Haygarth, is chiefly owing to an irregular sprouting of bone; and there exists a particular tendency of the edges of the joints to be extended by bony prolongations, often felt externally, and contributing to limit the motion of the part. There is frequent proneness to form loose pieces of solid consistence within the joint; and Dr. Adams has met with these very frequently, and in remarkable numbers, within the bursa of the olecranon. Numerous detached bursæ, entirely separate from the cavity of the joint, have been found about the joint affected; we understand, in the elbow, (p. 15;) and these, by desiccation, may become nodose in their characters. A crackling noise is very generally heard in rubbing the joint; and this occurs in every part of the body. The articulation finally changes its shape, and becomes much more bulky and irregular, independently of the nodosities. Dr. Adams has never met with the deposits of gouty matter. Of anchylosis, he has seen but one example, which was in the wrist. One would infer from his language that he inclined to think that Sir Benjamin Brodie, in mentioning the occurrence of this lesion, must have intended to use the word anchylosis in the sense which includes false anchylosis. Purulent deposits are rare, but Dr. Adams has met with them, in several instances, within the cavity of the affected joint.

*Prognosis.*—This, as far as relates to the prospect of a cure, appears, both by the observations of our author and of Dr. Haygarth, to be always unfavorable. The changes go on gradually increasing, with no intermission and little remission, during the remainder of life. If the disease be local, a well-managed treatment and favorable circumstances may enable the party to select some new employment suited to the condition of his members. But woe unto those in whom it appears as a general and constitutional affection, assailing many of the joints dispersed throughout the whole body. Their rest becomes imperfect, they become excessively sensitive to atmospheric changes, and little can be done for them in the way of remedial treatment. These ultimately form those unhappy cripples, with many limbs distorted, whom we meet as the permanent inhabitants of our almshouses. They are carried off in considerable proportion during the winter season, by dysentery, diarrhœa, and acute inflammations of the viscera, and sometimes by phthisis.

It would not appear, from the observations either of Dr. Adams or of Dr. Haygarth, that this affection, under favorable circumstances, shortens life, whether among the luxurious, who commit excess at the dinner-table, and avoid exercise, or among the poor, who suffer by cold. Dr. Adams believes that even the unhappy objects last described, if provided with the comforts and conveniences of life, clothed warmly, and enabled, though they may not be capable of walking, to take exercise of some description in the open air, appear to live as long as others of the same age. Dr. Haygarth knew no instance in which the nodosities and their attendant affections seemed to him to shorten life. The first individual he attended for this complaint lived to ninety-three years.

It will be observed that there is no mention among all this "articular rheumatism," of subsequent disease of the valves of the heart, with suffocation and death; nor is there, in fact, as already observed, any such throughout the whole volume.

*Diagnosis.*—On this important and difficult point Dr. Adams has furnished us with no separate recapitulation. It is only to be gathered, we apprehend, from the general description of the symptoms; and of these we have given the best abstract in our power. In the further special details of different joints, as they are affected by the disease, new characters certainly arise; but when we recollect his frequent amalgamations of his own cases with those of Dr. Haygarth, which were gouty, it is difficult perhaps to define the distinction between rheumatic and common gout in them. Other diagnostic rules will suggest themselves between the deformities of joints from this cause, and the fractures, dislocations, and other lesions which occur in surgery. On these Dr. Adams does not specially enlarge; leaving the diagnosis, as before, to be inferred from his detailed and very accurate descriptions, and his beautiful plates.

*Anatomical Changes.*—This very curious portion of our author's inquiries is also the richest. If the patient is cut off by some other agency, during the early stage of this affection, Dr. Adams predicts that we shall meet, besides the presence of an accumulation of synovia, with thickening of the ligaments and synovial membrane, and internal redness of the latter, with a great increase in size and color of the normal vascular tufts. We have a right to believe that Dr. Adams has seen dissections at this stage; but he does not assert it, or give any cases or plates to exemplify it.

With time, the fluid in the synovial membrane is reduced to about its ordinary amount. The capsule is then found thickened: in one instance this was the case to the extent of a quarter of an inch. It approached in appearance to the intervertebral substance. In some cases pieces of bone are found in its texture. There takes place, also, a removal of normal structures. The round ligament of the hip, and the intra-capsular part of the long tendon of the biceps, in the shoulder, in prolonged and severe cases, are generally removed; while the attached muscular fibres of the last-named flexor are not rendered useless, as the mutilated end of the tendon forms a firm adhesion to the edges of the groove between the tubercles of the humerus in which the cord naturally slides. The cartilages which incrust the ends of the bones are very frequently removed in part or altogether. The interarticular cartilages of the joint between the lower jaw and os temporis, of that between the sternum and the clavicle, and of the knee, are, in tedious and severe cases, generally removed entirely. In one subject, the semilunar cartilages were hypertrophied; and in another, partly ossified. The fibro-cartilaginous brims of the glenoid cavity of the shoulder and the cotyloid cavity of the hip, are liable to be removed by absorption. The lateral ligaments of ginglymoid joints are elongated.

A not unfrequent consequence of all this is looseness, and a capability of abnormous motion, extending to subluxation, and sometimes to luxation. Small movable masses, occasionally, as we have seen, discovered during life, are very frequently formed within the synovial membrane; occasionally in large numbers. One elbow-joint, made the subject of a plate, exhibited forty-five! These masses are cartilaginous, occasionally containing small pieces of bone, or incrusted with bone. They are generally attached by long, slender filaments, and occasionally connected together by a similar medium. In the remarkable instance recently mentioned, they were in groups or clusters. We do not observe that any were found absolutely detached. In one shoulder a large, abnormous cartilage in the form of a crescent embraced the anatomical neck of the humerus, and was attached with some firmness at its two extremities, thus strengthening the joint In some cases, abnormous pieces of bone were found deepening the cavities of arthrodial joints. Bright, vascular fimbriæ were, in some cases, very numerous.

The original bones were sometimes increased in size throughout their whole extent. Where thickness of bones was sawed through, the section exhibited unusual redness; but the most remarkable change in these organs was the conversion of that part of their articular surfaces from which the cartilage had been removed, into an ivory-like substance, with transverse ridges and hollows, following the direction of the most frequent motion of the joint, and fitting into a corresponding arrangement in the opposite bone. Where a ball-and-socket joint was thus affected, there was, consequently, a tendency to convert it into a ginglymus. The spherical or cylindrical surfaces, as of the upper extremity of the radius, at the ends of bones, were rendered oval; or the spherical surface sometimes replaced by a portion of a much larger sphere, diminishing the convexity.

*Treatment.*—Dr. Adams begins the subject with the inquiry whether the disease be inflammatory, and decides that it is so. With the modesty which characterizes the work throughout, he depends more upon the opinions of previous writers on the disease than on his own; and yet we could wish that he had analyzed and arranged his materials more fully, and given us some criticism on them. An anxious mind might experience difficulty in deciding what treatment he really recommends; and a prejudiced one might not be adequately influenced by opinions hinted at or expressed with so much diffidence. Our readers will see that the discussion of the inflammatory nature of the complaint is rather between two modes of viewing subjects of the class, two philosophies of pathology, than between the experience of different men on a practical problem. There is no doubt of the existence of many of the phenomena of inflammation in the cases, both before and after death.

Notwithstanding the unfavorable prognosis, Dr. Haygarth derived benefit from the warm-bath, even in very bad cases, as also from continued affusions of warm water, and from the repeated application of leeches. Sir Benjamin Brodie believes that much may be done at the very commencement of the disease. He recommends a very careful system of diet, including the very moderate use of animal food, a prohibition of fruits, acids, raw vegetables, and sugar, and the use of little or no fermented or spirituous liquor. The patient should take exercise daily so as to induce perspiration; and if this cannot be readily accomplished, he may, from time to time, take alterative doses of acetous extract of colchicum, combined with a small quantity of the mercurial pill, and use occasional purgatives. Moderate doses of potash or magnesia may be given, three or four hours after each of the principal meals, so as to neutralize any superabundant acid in the stomach. Soda should be carefully avoided, as tending to unite with uric acid, and form gouty concretions. Where the patient is depressed, as by the use of colchicum, a mixture of rhubarb, magnesia, and ginger may be given every night, with an active aperient at stated intervals, and very great benefit will often be derived from long perseverance in this procedure. If there be more than usual pain, leeches may be applied; and sometimes a light bandage, to limit motion, or even a light splint or two. Little permanent benefit, according to Sir B. Brodie, is to be expected, in general, from any local remedies; and he recommends them as palliatives. When the disease is confirmed, he reserves mercury and colchicum for special occasions. He believes that iodide of potassium is not wholly undeserving of reputation in the case; (grains ij...iij, twice a day, continued for several weeks.) One patient was benefited by the use of cod-liver oil internally and externally. These opinions of our old teacher, so well known and familiar to us as "Mr. Brodie," are well suited to the character of a great surgeon. Without presuming to say that no future discoveries or more extended experience will cause any of his directions or judgments to be superseded, they exhibit the idea of a man, profound in his elevated profession, and equally conscientious in the discharge of it; careful and laborious in the relief of the sick and injured, and scrupulous in making known the results of his observations, to enable others to go and do likewise. The value of these, future ages will be able to estimate. One who well knew Philip Syng Physick feels best prepared to do honor to Benjamin Brodie; who, after all his sovereign and countrymen have done to honor and distinguish him, has a still higher claim to grateful and praiseworthy remembrance than can be satisfied by queens and nations.

With this reserve of untarnished respect, we are tempted, by the very copiousness of Sir Benjamin's directions, to a comment or two, which we shall try to make in the love of truth. They remind us, first, of the gouty character of his cases; and of the circumstance that the state of things described by Dr. Adams is the consequence of two different sets of causes, distinctly implying two very different states of constitutional predisposition; all the difference, in iteration, between gout and rheumatism. That unfortunate word, diagnosis! Next, we hope to be excused for reminding our readers of the habit, prevailing extensively among the countrymen of the author whose opinions have been just recited, of considering the daily use of beer or wine, if not spirits, as a matter of course. Again, we presume that a more modern chemistry will hardly indicate much utility in avoiding soda, when there are always present such large supplies of this substance in the blood. (See *Lehmann*, Am. ed. i., pp. 546 and 590.) Another reflection may be thought harsh, and possibly unbecoming. We allude to the practice of referring medical formulæ to hospital or locally popular names, instead of making them common to the world by the ordinary means of publication. This we suppose to be the result of haste, or of the indolence of fatigue; but, if we dare ask the question, does it not *encourage the encouragement* of nostrums? If it do not bring business to "our shop," it may help to bring students to "our hospital;" a resource which the established and world-wide reputations in question render very unnecessary. "Dr. Gregory's Powder," for rhubarb, magnesia, and ginger, "sold" under that name by somebody; and "The

Chelsea Pensioner's Electuary," for a compound of sulphur, guaiacum, "et cetera"!

Dr. Watson is next quoted, (Lectures, third edition, 1848.) He advises flannel, warm climates, warm bathing, particularly with salt water, warm douches, vapor baths, hot-air baths, friction and shampooing; also turpentine, some animal oils, as cod-liver oil, which he includes among stimulants, guaiacum, iodide of potassium, and Dover's powder.

Dr. Adams states his own advice, without exactly summing up. In the sub-inflammatory stage, which we should call the subacute, the usual "treatment for chronic arthritis should be had recourse to." We are tempted to ask why, if the disease is not gouty, but rheumatic? He can report favorably of the use of cups and leeches, and of that of the Chelsea remedy already spoken of.* Anodynes afford considerable relief; though why, unless for persons habituated to opium, we should mix the watery extract of that substance with p. ipec. comp., we do not exactly see. It appears, also, that Dr. Adams has hardly been particular enough in pointing out the stage at which a disease, originally accompanied with so little pain, begins to require strong opiates. It is easy to imagine the necessity of it with those who have many joints diseased. Benefit has been derived from ammoniated tincture of guaiacum. Discrimination is important with regard to exercise. In the subacute stage of the commencement, rest is absolutely necessary; but at later periods, when stiffness is more characteristic than pain, some walking every day is desirable. It will improve the general health, and increase the flexibility of joints, and extend their power of motion. Dr. Adams's experience coincides with that of Messrs. Teissier and Bonnet, that prolonged total rest of joints is highly injurious to their usefulness; and here much experience coincides with them and him. We have not taken the time to examine the statements of these writers, beyond a few sentences extracted in Dr. Adams's Atlas. Dr. Adams agrees with them in finding that this error of management is calculated to determine the effusion of a sero-sanguineous fluid into the synovial sacs, the formation of false membranes, and erosion and thin-

---

* In the absence of the formula of Sir Benjamin's Hospital, we copy, from Jourdan, the anti-rheumatismal Electuary of Niemann's notes to the Pharmacopœia Batava; which we presume to be convenient and think neat:—

| | | |
|---|---|---|
| ℞.—Sulphuris | partes | lx. |
| Gum-resin, guaiac. et | | |
| Rhabarbari, āā | partes | vij. |
| Supertart. potass | " | xxx. |
| Nuc. myrist., fragrant. (Pereira.) | No. | j. |
| Mellis despumati | partes | ccclx. |

M. ft. electuarium s. a.

ning of the cartilages. Of thermal waters, he apprehends that experience is insufficient to decide in point of comparative usefulness in this affection; and deems that such will probably be the case till the disease shall have become more generally understood, and distinguished from gout and rheumatism properly so called, and until subsequent experience shall have been collected and recorded. This remark revives our regret for the want of a more explicit diagnosis. Finally, he renews his caution to attack the disease in its early stage.

We have been thus minute with the earlier part of the volume from judging it the most important. The remaining and larger portion (300 pages and a number of wood-cuts and plates) is occupied with the details of the effects of this disease upon the various joints of the body, and with the history of cases. A few cases are likewise related in the Atlas.

In the hip-joint, Dr. Adams does not approximate the disease under discussion to the old-fashioned *morbus coxarius* of young persons, which he considers scrofulous. When the present reviewer saw a number of these cases, scrofula was, as it still is, a decidedly rare disease in Philadelphia. We here mean the glandular disease, excluding all the affections of the eyes, ears, skin, etc., somewhat hypothetically, and on authority, taken to be scrofulous. We have never seen very clearly the difference, either in symptoms or morbid appearances, between that affection and other chronic inflammations of joints; not denying, with an equivalent hypothesis, that there may sometimes have been a tubercle in the case, within one of the bones concerned.

A *morbus coxæ senilis* is identified with the present disease by Dr. Adams, and also with allusions of Benjamin Bell, and descriptions by Sandifort, of Leyden, in 1793, and by Professor Smith, of Dublin. It has been exhibited and lectured on for many years in the hospitals of the last-named city. Dr. Adams thinks that cases of this kind have been found in dissection, and taken for cures of fractures of the neck of the thigh-bone within the capsule. The well-known obscurity and difficulty of diagnosis in injuries in the vicinity of the hip-joint, certainly leave ample room for future discovery, and a strong moral motive for inquiry.

In the shoulder the amount of destruction seems proportionally much greater. The head of the bone is often enlarged to part of a much larger sphere, a cause which gave rise to difficulty in Lisfranc's mode of amputation; the fibro-cartilaginous edges of the socket are removed, and a very large and totally abnormous joint is formed. In some cases the joint subsides to a lower level. We have mentioned the destruction of the upper part of the long tendon of the biceps, which seems to be one of the earliest of the severer changes. The case of "partial luxation of the shoulder-joint forward and inward," described from a dissecting-room in Sir Astley

Cooper's work, is believed, by Dr. Adams, to be one of the affection under consideration; and Dr. Adams's figures go far to confirm this conviction. One hesitates much to accuse Sir Astley Cooper of an error in diagnosis; but the interests of science and humanity demand the truth, and Sir Astley was mortal.

Our limits compel us to abridge much that is highly interesting and useful, and we have chosen to do so principally in this part of the work. Under the article shoulder, are found analyses of a variety of published cases. The elbow is principally remarkable for multiplicity of loose cartilages and abnormous bursæ. In the knee, we have generally some pain in the inner condyle and in the tibia, with some heat and swelling of the joint, the patient walking for years. The knee inclines inward, and the foot is slightly everted; the fluctuating swellings of chronic synovitis are perceived, and occasionally accompanied by a swelling, about the size of a hen's egg, in the ham. The patella is liable to be thrown over the outer condyle, as in dislocation by violence. Roughness is felt, and a crepitating noise heard in flexing and extending the knee. The patella becomes broader than natural, and bony ridges are felt on both sides of the sulcus in which it slides. The knee is particularly subject to numbers of little cartilaginous bodies, like melon-seeds, attached by slender shreds, best described by Rokitansky; and to bony vegetations round the edges of the joint, growing from the femur. Various other changes, very curious and interesting, we are obliged to pass over. The distortions of the wrist and hand are very frightful and very various. These affections are more numerous here than in any other part of the body. The difference between local and constitutional cases is very marked here, the constitutional cases affecting both wrists at once. The most extraordinary distortions are produced, including dislocations. Details of the disease are given as it appears in the ankle, foot, articulation of the lower jaw, and sterno-clavicular, and acromio-clavicular articulations; and to illustrate those of the cervical vertebræ there are four cuts.

It is time now, however, to look toward a close. On summing up, there is no doubt of the justly high rank and great value of the work before us. It is a production in morbid anatomy, much called for, in a department inadequately explored, and the labor is rewarded by a large accession of knowledge. It is also the more valuable in the British schools, in which morbid anatomy has been by no means so much cultivated as in some others, or as its importance may demand. At the same time we candidly say that our author appears to us deficient in the art of bookmaking, not merely in the formal sense, but in one very real. He has brought together what it has cost the labor of so many observing and reflecting minds to separate; he has once more confounded gout and rheumatism together, and furnished a new pretence to those who find a shelter in obscurity and indefiniteness. As in the case of the common or the doubtful quantity of a Latin syllable, there is a real and important distinction between the two things; yet it is a great saving of trouble to confuse them, and a mind imperfectly informed can find comfort in the obscurity. It is well sheltered, too, against public opinion. The idea of such a mixture is an old one, and is now common among the people; hence, to a practitioner who is really not well enough informed on the subject, who is not ready at argument, or who is of a timid disposition, and fearful of discussion, lest it betray some weak point about him, it may be convenient to take shelter in a doubt which is taken for acknowledged among those with whom he practices. Unfortunately, however, this has an injurious influence on the profession. The argument that medicine is full of doubts, backed as it is by so many satirical poets, and by almost every petty pretender to the character of a philosopher, is always a favorite one with the quacks. "If your own science, as you claim it, is full of doubts and obscurities, what right have you to censure Thompsonians, Homœopathians and others for alleged errors of theory?" They then lay their nauseous claim to be rival "schools" in medicine, and advance the mendacity of alleged "successes."

The fact is that to draw the distinction between truth and error, and between uncertainty and reasonable human proof, is the first and highest duty of a teacher. In this venerated position we place both those who, like our author, extend the limits of useful science, and those whose labor it is to teach what is already known, and methodize it by systematic treatises or public lectures. We therefore regret that Dr. Wood, in a passage already quoted, leaves this point as doubtful, and seems to depend on future usage to settle whether the affection which results alike from the radically and widely different sources of gout and rheumatism, shall be treated as a mixture, or rather as an indefinite obscurity between them. It appears to the present writer that this professor's choice has led, or amounted to a fault; and that the reader had claims, not only to a statement of an apparently unsettled point, but to the decision of an eminent and celebrated teacher thereupon; not merely to the information that such a discrepancy remained in general usage, and appeared to have been revived by Dr. Adams, but to the judgment of his master which was the correct view.

Nor is even this all. It is not a mere looseness of expression in grouping together a set of severe phenomena, equally produced by two sets of causes, requiring totally different, and in some respects opposite, modes of prevention and cure. This is not the popular confusion. The impression among the people is, that gouty rheumatism is a mixture of the two things together, and that it pervades the whole body. This is the natural meaning of this very incorrect phrase, as well as of rheumatic gout; not common results from two causes, but a mixture of two results. Hence an excuse for all kinds of blindness and experimental management, a fear of any persevering treatment lest another half of the disease be neglected, increased hopelessness of the cure from the alleged complication in the malady, and a readiness, at any moment, to "try a little," never sufficiently to ascertain the truth, the resource of another physician or practitioner, of any imposture, of any obliging neighbor's prescription, of any common drug of worn-out reputation, or of any plant; all taken at random, without method, or any real hope of cure. Hence if a physician should be guilty of giving way to the continuance of the error we have above exposed, he is himself punished in the long run, and involves his unoffending friends.

Opposition to this cannot be blamed as selfish or interested, when it consists in an increased effort after certainty and accuracy, and after scrupulousness in distinguishing between that portion of medicine which has gratified the malice of French theatrical wits of another age, and of small philosophers, and men of the world in ours, styling their art an *ars conjectandi*, from that which is set forth and sustained by a conscientious, well-informed, and careful study, as matter of truth and real science.

